# Muscle fiber capillarization as determining factor on indices of insulin sensitivity in humans

**DOI:** 10.14814/phy2.13278

**Published:** 2017-05-26

**Authors:** Tim Snijders, Joshua P. Nederveen, Lex B. Verdijk, Alfons J. H. M. Houben, Gijs H. Goossens, Gianna Parise, Luc J. C. van Loon

**Affiliations:** ^1^ Department of Human Biology and Movement Sciences NUTRIM School of Nutrition and Translational Research in Metabolism Maastricht University Medical Center+ Maastricht The Netherlands; ^2^ Department of Kinesiology McMaster University Hamilton Ontario Canada; ^3^ Department of Internal Medicine CARIM School for Cardiovascular Diseases Maastricht University Medical Center+ Maastricht The Netherlands

**Keywords:** Aging, oral glucose tolerance, skeletal muscle

## Abstract

To investigate the association between muscle fiber capillarization and indices of insulin sensitivity in healthy older adults. A skeletal muscle biopsy was taken from the *m*. *vastus lateralis* of 22 healthy (nondiabetic) male older adults. In addition, all participants underwent an Oral Glucose Tolerance Test (OGTT). Muscle fiber capillarization was assessed by immunohistochemistry. Participants were divided into a group with relatively low (LOW) or high (HIGH) muscle fiber capillarization (capillary‐to‐fiber perimeter exchange (CFPE) index), based on the median value for the entire group. All participants were healthy, nonobese, and had a normal glucose tolerance, according to the individual OGTT results. Whereas no differences in blood glucose concentrations were observed between groups during the OGTT, the postprandial increase in plasma insulin concentrations was significantly greater in the LOW compared to the HIGH muscle fiber capillarization group (*P* < 0.05). Skeletal muscle fiber capillarization may determine insulin sensitivity in humans.

## Introduction

Skeletal muscle capillarization may regulate a number of physiological processes as the number of capillaries can be a limiting factor in the delivery of oxygen, substrates, and/or hormones (Gudbjornsdottir et al. 2003). Transcapillary transport of insulin is an important determinant of glucose uptake in skeletal muscle tissue (Yang et al. 1994; Barrett et al. 2009). Consequently, a lower level of muscle fiber capillarization may be an important determinant of glucose intolerance or whole‐body insulin resistance. In accordance, previous studies report a relationship between skeletal muscle fiber capillarization and insulin sensitivity in young (Lillioja et al. 1987) and older individuals (Marin et al. 1994; Hedman et al. 2000; Prior et al. 2009). However, the subjects' characteristics in previous studies are quite heterogeneous with respect to age, body weight/composition, training status, and disease state, which may drive the observed associations (Lillioja et al. 1987; Marin et al. 1994; Hedman et al. 2000; Prior et al. 2009). Whether muscle fiber capillarization modulates glucose homeostasis in a more homogeneous group of healthy, nondiabetic, older adults has so far not been established. In this study, we investigated whether glucose tolerance differs between participants with a relatively low versus high skeletal muscle fiber capillarization in healthy, nondiabetic, older adults. We hypothesized that a low skeletal muscle fiber capillarization is associated with impaired insulin sensitivity.

## Research Design and Methods

Twenty‐two healthy male older adults (age: 70 ± 6 year, height: 1.76 ± 0.06 m, BMI: 27.3 ± 2.6 kg·m^−2^) were included in this study. Participants were excluded in case of (silent) cardiac, peripheral vascular disease or orthopedic disorder. All participants were recruited in local area of Maastricht University (the Netherlands) via an advertisement in local newspapers and had not participated in any structured exercise training program in the past 5 years and were all living independently. All participants were informed of the nature and possible risks of the experimental procedures before their written informed consent was obtained. This study was approved by the Medical‐Ethical Committee of Maastricht University and complied with the guidelines set out in the Declaration of Helsinki. This study is part of a greater study investigating the impact of nutrition and exercise on skeletal muscle health in older adults (Leenders et al. 2013).

## Procedures and Analyses

All participants arrived at the laboratory in the morning following an overnight fast, after which a single muscle biopsy was taken from the *m*. *vastus lateralis*. After local anesthesia was induced in the skin, a percutaneous needle biopsy sample (50–80 mg) was collected from the vastus lateralis muscle, approximately 15 cm above the patella. Any visible nonmuscle tissue was removed immediately, and biopsy samples were embedded in Tissue‐Tek (Sakura Finetek, Zoeterwoude, the Netherlands), frozen in liquid nitrogen‐cooled isopentane, and stored at −80°C until further analyses. Next, a standard Oral Glucose Tolerance Test (OGTT) was performed, as described previously (Leenders et al. 2013). Blood samples were taken from an antecubital vein at baseline (*t* = 0 min), and after 30, 60, 90, and 120 min following glucose ingestion. The participants were instructed to refrain from strenuous physical activity for at least 3 days prior to the test day. To assess habitual physical activity, participants completed a 2‐day physical activity diary 1 week prior to the test day. Whole‐body and regional fat and fat‐free mass were determined by DXA scan (Discovery A, QDR Series; Hologic, Bradford, MA). Leg strength was assessed by performing a one‐repetition maximum (1RM) strength test on a leg press apparatus (Leenders et al. 2013). All participants completed the entire research protocol, there were no drop‐outs.

### Blood analyses

Plasma insulin and glucose concentrations were determined by using an Insulin RIA Kit (LINCO Research Inc., St Charles, MO) and COBAS FARA analyzer (Uni Kit III; Roche, Basel, Switzerland) and a test kit from ABX Diagnostics (Montpellier, France), respectively. Blood HbA1c contents were analyzed by high‐performance liquid chromatography (Bio‐Rad Variant II 4, Munich, Germany). Indices of whole‐body insulin sensitivity and/or oral glucose tolerance were assessed by fasting blood glucose and insulin concentrations using the oral glucose insulin sensitivity (OGIS) index and the insulin sensitivity index (ISI) were calculated from the data derived from the OGTT, as described previously (Gutt et al. 2000; Mari et al. 2001).

### Immunohistochemistry

Samples were stained with antibodies against myosin heavy‐chain type I (clone A4.951 (slow isoform), neat; DSHB); laminin (1:1000; Abcam ab11575, Abcam, Cambridge, MA) and CD31 (ab28364 1:30, Abcam, Cambridge, MA). For immunofluorescent detection, secondary antibodies used were as follows: myosin heavy‐chain type I (clone A4.591) (goat antimouse Alexa Fluor 488, 1:500, Invitrogen); laminin (goat antirabbit Alexa Fluor 488, 1:500, Invitrogen); and CD31 (goat antirabbit Alexa Fluor 647, 1:500, Invitrogen, Molecular Probes, Carlsbad, CA). Histochemical methods were adapted from previous published methods (Nederveen et al. 2016; Snijders et al. 2017). Slides were viewed with the Nikon Eclipse Ti Microscope (Nikon Instruments, Inc), equipped with a high‐resolution Photometrics CoolSNAP HQ2 fluorescent camera (Nikon Instruments, Melville, NY). Images were captured and analyzed using the Nikon NIS Elements AR 3.2 software (Nikon Instruments, Inc.). The quantification of muscle fiber size and type distribution was performed on 176 ± 19 and 145 ± 19 type I and type II muscle fibers per subject, respectively. In addition, CFPE index was determined on ≥50 muscle fibers per subject. CPFE index was calculated as described previously (Hepple et al. 1997).

### Statistics

Data are expressed as means±SD. Based on the median value for the entire group, participants were divided into two equal groups with relative LOW (mean: 5.2 ± 0.6 capillaries·1000 *μ*m^−1^) or HIGH (mean: 7.0 ± 0.9 capillaries·1000 *μ*m^−1^) CFPE index. All Kolmogorov–Smirnov test was used to check for normality. All dependent parameters were normally distributed according to Kolmogorov–Smirnov test, with the exception of age and mean energy expenditure. For all normally distributed parameters, an independent samples t test was used to examine differences, body composition, muscle strength, muscle fiber size, and blood profile between the LOW and HIGH groups. A nonparametric Mann–Whitney U test was performed to assess difference in age and mean energy expenditure between the LOW and HIGH group. OGTT results were analyzed using repeated measures ANOVA with time (T0, T30, T60, T90, and T120 min) as within‐subject factor and group (LOW vs. HIGH) as between‐subject factor. Bonferroni correction was applied to correct for multiple testing. Pearson correlation analyses was used to established whether mixed muscle fiber CFPE index was associated with different indices of glucose homeostasis/insulin sensitivity, fiber type distribution, and whole‐body fat mass in the entire group of subjects. Significance was set at *P* < 0.05. Calculations were performed using SPSS version 21.0 (Chicago, IL).

## Results

### Subjects' characteristics

No significant differences were observed between LOW and HIGH for age (71 ± 7 vs. 69 ± 5 year), height (1.76 ± 0.05 vs. 1.76 ± 0.07 m, respectively), whole‐body lean mass (62.8 ± 3.8 vs. 59.8 ± 5.7 kg, respectively), leg lean mass (19.7 ± 1.2 vs. 19.3 ± 1.9 kg, respectively), and 1RM leg press strength (212 ± 28 vs. 207 ± 21 kg, respectively). In addition, type I and type II muscle fiber size (average per fiber) were comparable between the LOW (5907 ± 1362 and 5130 ± 935 *μ*m^2^, respectively) and the HIGH (5759 ± 963 and 5171 ± 1295 *μ*m^2^, respectively) groups. Interestingly, the percentage of type I muscle fibers was significantly lower in the LOW (45 ± 4%) compared with the HIGH (64 ± 5%) group (*P* < 0.01). Body mass index (BMI) was significantly higher in the LOW (28.9 ± 2.0 kg·m^−2^) compared with the HIGH (25.8 ± 2.4 kg·m^−2^) group (*P* < 0.01). In addition, whole‐body fat mass was significantly higher in the LOW (23.1 ± 4.8 kg) compared with the HIGH (17.2 ± 5.0 kg) group (*P* < 0.05). Habitual physical activity level, expressed as mean energy expenditure per day, did not differ between groups (LOW: 1.57 ± 0.27 vs. HIGH: 1.48 ± 0.16 MET·d^−1^, respectively). No differences were observed in HbA1c between groups (LOW: 5.5 ± 0.5 vs. HIGH 5.5 ± 0.4%, respectively).

### Oral glucose tolerance test

**Figure 1:**
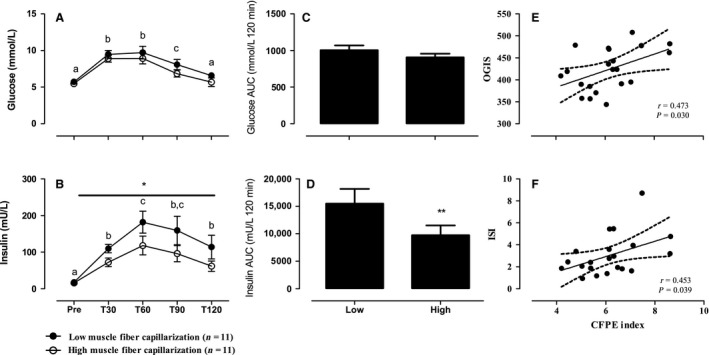
Plasma glucose (**A** and **C**), insulin concentrations (**B** and **D**), and insulin sensitivity indices (**E** and **F**) during an oral glucose tolerance test (OGTT) in healthy older adults with a relative low and high level of muscle fiber capillarization. Data are presented as Means±SEM. Low: relative low muscle fiber capillarization is based on capillary‐to‐fiber exchange (CFPE) index. High: relative high muscle fiber capillarization based on CFPE index. * Significant effect of group *P* < 0.05. ** Significantly different compared with Low *P* < 0.05. Different letters indicate significant differences from one another. Pearson correlation between CFPE and Oral Glucose Insulin sensitivity (OGIS) (**E**) and Insulin Sensitivity Index (ISI) (**F**).

At baseline, no differences in fasting plasma glucose (5.8 ± 0.5 vs. 5.4 ± 0.5 mmol/L, respectively) and insulin (18.9 ± 4.9 vs. 14.3 ± 4.3 mU/L, respectively) concentrations were found between the LOW and HIGH group, respectively. In response to glucose ingestion, plasma glucose levels increased significantly over time (main effect of time *P* < 0.0001) and returned back to baseline level at *t* = 120 min, with no differences between the groups (Fig. 1A). In agreement, plasma glucose area under the curve did not differ between groups (Fig. 1C). Based upon the individual plasma glucose values from the OGTT all participants were normal glucose tolerant. Plasma insulin concentrations increased significantly over time, with peak levels being reached at *t* = 60 min, in both groups (Fig. 1B). Although no significant time x group interaction (*P* = 0.125) was observed, we did find a main effect for group (*P* < 0.05), indicating that overall insulin concentrations were higher in the LOW versus the HIGH group (Fig. 1B). In accordance, plasma insulin concentrations expressed as area under the curve were significantly higher in the LOW versus the HIGH group (Fig. 1D). In addition, ISI and OGIS were significantly lower in the LOW (ISI: 2.0 ± 0.8 and OGIS: 395 ± 42, respectively) compared with the HIGH (ISI: 4.0 ± 2.1 and OGIS: 450 ± 38, respectively) group (both *P* < 0.05). A significant positive correlation was observed between mixed CFPE index and OGIS (*r *=* *0.473, *P* = 0.030; Fig. 1E) and ISI (*r *=* *0.453, *P* = 0.039; Fig. 1F) in the complete group of subjects. Indicating that greater muscle fiber capillarization is associated with greater insulin sensitivity. Furthermore, the proportion of type I muscle fibers was significantly correlated with OGIS (*r *=* *0.461, *P* = 0.036), ISI (*r *=* *0.477, *P* = 0.029), and whole‐body fat mass (*r *=* *−0.591, *P* < 0.01). Finally, we observed a significant correlation between whole‐body fat mass and CFPE index (*r *=* *−0.618, *P* < 0.01), OGIS (*r *=* *−0.706, *P* < 0.01), and ISI (*r *=* *−0.684, *P* < 0.01).

## Discussion

This study shows that relative low muscle fiber capillarization is associated with indices of lower whole‐body insulin sensitivity in healthy older adults.

Previous studies have demonstrated a relationship between muscle fiber capillarization and insulin sensitivity (Lillioja et al. 1987; Marin et al. 1994; Hedman et al. 2000; Prior et al. 2009). However, these studies included participants from various age categories, body composition (lean and obese), and/or disease states (healthy, type II diabetes patients, chronic stroke patients), all of which may have confounded this association (Lillioja et al. 1987; Marin et al. 1994; Hedman et al. 2000; Prior et al. 2009). We confirm previous studies showing that low muscle fiber capillarization is associated with lower whole‐body indices of insulin sensitivity. However, we show for the first time that this relationship is also present in a relative homogeneous group with respect to age, disease state, lean tissue mass, muscle strength, fiber size, and/or HbA1c level. To reach similar blood glucose levels, the postprandial increase in insulin concentrations at *t* = 60 min was almost twofold higher in the LOW versus the HIGH group (Fig. 1B). This was further supported by significantly higher insulin AUC during the 2 h OGTT, and lower whole‐body insulin sensitivity indices (ISI and OGIS) in the LOW group. Despite the absence of differences in lean mass and/or fiber size between groups the level of capillarization was accompanied by postprandial insulin levels. Interestingly, the proportion of type I muscle fibers was significantly lower in the LOW (45 ± 4%) compared with the HIGH (64 ± 5%) group. In addition, a positive correlation was observed between the proportion of type I muscle fibers and indices of insulin sensitivity (e.g., OGIS and ISI). This is in line with previous reports showing a relationship between fiber type distribution and insulin sensitivity (Lillioja et al. 1987; Stuart et al. 2013). Due to their oxidative nature, type I muscle fibers are typically associated with a greater number of capillaries than type II muscle fibers. With aging type II muscle capillarization has been shown to decrease significantly (Groen et al. 2014; Nederveen et al. 2016). As muscle capillaries are frequently shared between different muscle fiber types, a higher percentage of type I muscle fibers will also likely result in an enhanced perfusion of type II muscle fibers subsequently increasing the overall perfusion capacity, and possible insulin sensitivity, of the entire skeletal muscle bundle. The relationship between fiber‐type composition and whole‐body fat mass observed might suggest that these are both part of a more generalized syndrome resulting in a decrease in insulin sensitivity and abnormal glucose tolerance.

Although in this study all subjects were considered nonobese (BMI <30 kg·m^−2^), we do observe a small, but significant, difference in BMI and whole‐body fat mass between the LOW and HIGH group. Furthermore, we observe a negative association between whole‐body fat mass and indices of insulin sensitivity and CFPE index. Although muscle fiber capillarization, fat mass, and insulin sensitivity are clearly interconnected, a previous study with a larger sample size has already demonstrated that while fat mass and muscle fiber capillarization are related, they both appear to have independent effects on whole‐body insulin sensitivity (Lillioja et al. 1987).

Although this study does not establish causality and we should not ignore the small (but significant) difference in BMI (fat mass) between the two groups, we speculate that capillary networks may be of more relevance for insulin‐induced postprandial glucose uptake when compared with the absolute amount of lean mass. Therefore, future studies should be aware that interindividual differences in muscle fiber capillarization, in a homogeneous group of (healthy) older adults, may be a decisive factor in a number of physiological processes involving the delivery of oxygen, substrates, and/or hormones to skeletal muscle.

In conclusion, the present findings suggest that skeletal muscle tissue capillarization represents an important factor in glucose tolerance, and may determine whole‐body insulin sensitivity in the older population.

## Conflict of Interest

No potential conflicts of interest relevant to this article were reported.

## References

[phy213278-bib-0001] Barrett, E. J. , E. M. Eggleston , A. C. Inyard , H. Wang , G. Li , W. Chai , et al. 2009 The vascular actions of insulin control its delivery to muscle and regulate the rate‐limiting step in skeletal muscle insulin action. Diabetologia 52:752–764. 19283361 10.1007/s00125-009-1313-z PMC2704146

[phy213278-bib-0002] Groen, B. B. , H. M. Hamer , T. Snijders , vanKranenburg J. , D. Frijns , H. Vink , et al. 2014 Skeletal muscle capillary density and microvascular function are compromised with aging and type 2 diabetes. J. Appl. Physiol. (1985) 116:998–1005. 24577061 10.1152/japplphysiol.00919.2013

[phy213278-bib-0003] Gudbjornsdottir, S. , M. Sjostrand , L. Strindberg , J. Wahren , and P. Lonnroth . 2003 Direct measurements of the permeability surface area for insulin and glucose in human skeletal muscle. J. Clin. Endocrinol. Metab. 88:4559–4564. 14557422 10.1210/jc.2003-030434

[phy213278-bib-0004] Gutt, M. , C. L. Davis , S. B. Spitzer , M. M. Llabre , M. Kumar , E. M. Czarnecki , et al. 2000 Validation of the insulin sensitivity index (ISI(0,120)): comparison with other measures. Diabetes Res. Clin. Pract. 47:177–184. 10741566 10.1016/s0168-8227(99)00116-3

[phy213278-bib-0005] Hedman, A. , L. Berglund , B. Essen‐Gustavsson , R. Reneland , and H. Lithell . 2000 Relationships between muscle morphology and insulin sensitivity are improved after adjustment for intra‐individual variability in 70‐year‐old men. Acta Physiol. Scand. 169:125–132. 10848642 10.1046/j.1365-201x.2000.00722.x

[phy213278-bib-0006] Hepple, R. T. , S. L. Mackinnon , J. M. Goodman , S. G. Thomas , and M. J. Plyley . 1997 Resistance and aerobic training in older men: effects on VO2peak and the capillary supply to skeletal muscle. J. Appl. Physiol. (1985) 82:1305–1310. 9104869 10.1152/jappl.1997.82.4.1305

[phy213278-bib-0007] Leenders, M. , L. B. Verdijk , L. Van der Hoeven , J. Van Kranenburg , R. Nilwik , W. K. Wodzig , et al. 2013 Protein supplementation during resistance‐type exercise training in the elderly. Med. Sci. Sports Exerc. 45:542–552. 22968306 10.1249/MSS.0b013e318272fcdb

[phy213278-bib-0008] Lillioja, S. , A. A. Young , C. L. Culter , J. L. Ivy , W. G. Abbott , J. K. Zawadzki , et al. 1987 Skeletal muscle capillary density and fiber type are possible determinants of in vivo insulin resistance in man. J. Clin. Invest. 80:415–424. 3301899 10.1172/JCI113088 PMC442253

[phy213278-bib-0009] Mari, A. , G. Pacini , E. Murphy , B. Ludvik , and J. J. Nolan . 2001 A model‐based method for assessing insulin sensitivity from the oral glucose tolerance test. Diabetes Care 24:539–548. 11289482 10.2337/diacare.24.3.539

[phy213278-bib-0010] Marin, P. , B. Andersson , M. Krotkiewski , and P. Bjorntorp . 1994 Muscle fiber composition and capillary density in women and men with NIDDM. Diabetes Care 17:382–386. 8062604 10.2337/diacare.17.5.382

[phy213278-bib-0011] Nederveen, J. P. , S. Joanisse , T. Snijders , V. Ivankovic , S. K. Baker , S. M. Phillips , et al. 2016 Skeletal muscle satellite cells are located at a closer proximity to capillaries in healthy young compared with older men. J. Cachexia Sarcopenia Muscle 7:547–554. 27239425 10.1002/jcsm.12105 PMC4864218

[phy213278-bib-0012] Prior, S. J. , M. J. McKenzie , L. J. Joseph , F. M. Ivey , R. F. Macko , C. E. Hafer‐Macko , et al. 2009 Reduced skeletal muscle capillarization and glucose intolerance. Microcirculation 16:203–212. 19225985 10.1080/10739680802502423 PMC2990692

[phy213278-bib-0013] Snijders, T. , J. P. Nederveen , S. Joanisse , M. Leenders , L. B. Verdijk , vanLoon L. J. , et al. 2017 Muscle fibre capillarization is a critical factor in muscle fibre hypertrophy during resistance exercise training in older men. J. Cachexia Sarcopenia Muscle 8:267–276. 27897408 10.1002/jcsm.12137 PMC5377411

[phy213278-bib-0014] Stuart, C. A. , M. P. McCurry , A. Marino , M. A. South , M. E. Howell , A. S. Layne , et al. 2013 Slow‐twitch fiber proportion in skeletal muscle correlates with insulin responsiveness. J. Clin. Endocrinol. Metab. 98:2027–2036. 23515448 10.1210/jc.2012-3876 PMC3644602

[phy213278-bib-0015] Yang, Y. J. , I. D. Hope , M. Ader , and R. N. Bergman . 1994 Importance of transcapillary insulin transport to dynamics of insulin action after intravenous glucose. Am. J. Physiol. 266:E17–E25. 8304440 10.1152/ajpendo.1994.266.1.E17

